# Are policy initiatives aligned to meet UNAIDS 90-90-90 targets impacting HIV testing and linkages to care? Evidence from a systematic review

**DOI:** 10.1371/journal.pone.0216936

**Published:** 2019-06-05

**Authors:** Nicolaos Karatzas, Trevor Peter, Sailly Dave, Clare Fogarty, Nandi Belinsky, Nitika Pant Pai

**Affiliations:** 1 Division of Clinical Epidemiology, Research Institute of the McGill University Health Centre, Montreal, Quebec, Canada; 2 Clinton Health Access Initiative, Gaborone, Botswana; 3 Department of Medicine, McGill University, Montreal, Quebec, Canada; NPMS-HHC CIC / LSH&TM, UNITED KINGDOM

## Abstract

**Background:**

The Joint United Nations Programme on HIV/AIDS (UNAIDS) Fast-Track initiative seeks to eliminate AIDS as a health threat by 2030, with its focus on UNAIDS 90-90-90 targets. Effective policies and programs, if scaled nationally, have the potential to generate a greater impact on HIV control, yet a synthesis of successful HIV policies/programs aligned to the targets is currently unavailable. To fill this gap, we conducted a systematic review to evaluate successful HIV policies and programs to direct future interventions.

**Methods:**

For the period 2007–2018, we searched 8 databases and classified eligible studies by country income level, UNAIDS targets, intervention type, and reported outcomes. Study outcomes were classified as per UNAIDS targets; proportionally: 90% target 1, 81% target 2, and 73% target 3.

**Results:**

We retrieved 5201 citations and a final set of eight studies on policies. Break up by income: three (38%) from high income, one (12%) from middle income and four (50%) from low income. Break up by outcomes reported: 36% (4/11) focused on HIV testing, 46% (5/11) on antiretroviral therapy initiation, and 18% (2/11) on viral suppression. Across studies, UNAIDS targets were met in high-income countries, where policies and guidelines were adhered to, whereas in low and middle-income countries, non-adherence led to failure to reach the targets. Targets were also met when country infrastructure supported a targeted program and stakeholders were actively engaged.

**Conclusions:**

From the studies identified, we deduced a clear, positive correlation between implementation of policies and programs that resulted in an increase in patient awareness and an increase in partner notification with services that encouraged them, and together these resulted in increasing testing rates, and deployment of linkage/retention programs that improved retention in care. An analysis of these studies also suggests that policies, combined with the scale-up incentives, are needed to change the status quo. Incentives to improve the targets must exist; performance incentives at the health care worker level and country level incentives that could transform the nature of care. Given the complexity in reporting of targets, a one size fits all model is not a feasible option. However, the policies created a strong framework to shape future interventions.

## Introduction

### Rationale

In October of 2014, the Joint United Nations Programme on HIV/AIDS (UNAIDS) and the World Health Organization (WHO) announced the 90-90-90 Fast-Track initiative to end the global HIV/AIDS pandemic as a public health threat by 2030 [1]. Countries pledged to collaboratively strive to achieve a “90% reduction of HIV incidence and AIDS related mortality from 2010 through 2030” [2]. In order to achieve this goal, UNAIDS launched the 90-90-90 targets [3]. Each 90 of the UNAIDS targets refers to a diagnosis of HIV, the initiation into care, and achieving viral suppression respectively. UNAIDS definitions imply that each target is looking at 90% of the previous target in the cascade. UNAIDS has made it clear that this goal will not be achieved solely through these targets and that additional targets will be required [2].

Global HIV/AIDS statistics reveal that, despite these efforts, as of 2017, only 38% of people living with HIV/AIDS were virally suppressed, yet the first 90 target is close to 75% [2, 4].

Several key factors play a role in global implementation of UNAIDS targets that are good metrics for quantifying the HIV testing and care cascade; it is mistakenly believed that a significant factor in achieving success is a country’s income status.

In low and middle-income countries, significant barriers impede HIV prevention, treatment, and control [5]. Such barriers include underfunded public health systems with weak primary care continuums, the marginalization of impacted communities, poverty, poor health literacy and consequent lack of awareness and education regarding HIV/AIDS, and above all, social stigma and discrimination of people living with HIV/AIDS.

However, although these barriers differ in high income countries, marginalized populations face similar stigmatization issues in accessing care. A considerable amount of work is still required to achieve the goal of HIV/AIDS elimination; specifically, putting in place effective policies and programs that are personalized to each country. Therefore, reaching the UNAIDS 90-90-90 targets and ending the health threat of HIV/AIDS relies heavily on evidence-based, proven interventions that effectively engage, screen, link and retain previously untested HIV+ individuals to care. Despite pronounced efforts and resources, success has fallen short for many high-income countries, with target achievement rates below the 90 percent mark [6].

Amongst high-income countries, Scandinavia continues to set high standards as Sweden recently announced the national achievement of all three 90-90-90 goals [7, 8]. Middle-income countries have also reported important declines in prevalence, illustrating the impact that concerted care efforts can have in reducing HIV burden where resources are limited and seldom available. Efforts must continue at every step to mend the leaky cascade of HIV care wherein people are lost to follow up.

In addition to policy guidelines, UNAIDS-led analyses have shown that certain factors predict success in achieving these targets [9]; namely: a) the epidemic stage within the country, b) the percentage of crucial populations affected, c) the prevalence of epidemic in those populations, d) incidence rate of new HIV infections, e) burden of prevailing infections, f) costs of treatment, g) available funding, and h) the capacity of health systems to influence a country’s progress [9]. Policies together with funded programs need to be implemented, thereby employing a “personalized” approach to further build on the successes. This analyses reflects that the targets alone do not suffice, yet are a good starting point to uncover gaps in the implementation of the care continuum.

The Global HIV Policy Watch, a crowd-sourced bank, allows one to research different policies implemented across the globe. We observed that a gap between policy and programs and field implementation directly impacts implementation. To fill this knowledge gap in understanding national policies and programs that were deemed successful in reaching UNAIDS 90-90-90 targets, and to highlight successes per UNAIDS targets, and we conducted a systematic review.

### Objectives

Our review aims to address the following:

1. To review policies and programs that have implemented and demonstrated progress towards UNAIDS 90-90-90.
2. To focus on creating a compendium of policies and programs available globally and assess their success based on both their progress and rate of progress.

## Methods

To meet these objectives, we systematically and critically appraised and reviewed evidence on all policy-based initiatives, and also collated data aligned to UNAIDS targets (wherever available). We followed The Preferred Reporting Items for Systematic Reviews and Meta-Analyses (PRISMA) to conduct this systematic review (see S1 Checklist).

### Protocol and registration

We did not register with Prospero.

### Data sources and searches

We searched eight databases (BIOSIS, CINAHL, Cochrane Library, EMBASE, Global Index Medicus, LILACS, OVID, PubMed) and three conferences (CROI, IAS, and ASLM) for articles published between the period 2007–2018.

### Search strategy

We used keywords such as UNAIDS, 90-90-90, HIV and AIDS. An example of the search string used for PubMed is as follows: (("HIV"[MeSH Terms] OR "HIV"[All Fields]) OR ("acquired immunodeficiency syndrome"[MeSH Terms] OR ("acquired"[All Fields] AND "immunodeficiency"[All Fields] AND "syndrome"[All Fields]) OR "acquired immunodeficiency syndrome"[All Fields] OR "aids"[All Fields])) AND (“UNAIDS”[All Fields] AND/OR “90-90-90”[All Fields] AND/OR (“youth” OR “adults”) AND “viral load”).

Choosing to search for the UNAIDS 90-90-90 in our search fields limited our search to 2014 or after as this represents the implementation year of the UNAIDS targets.

Mention of “policy interventions”, in the effort to end the HIV/AIDS pandemic, began to be referenced in 2007. For this reason, 2007 was chosen as a starting date for our search string.

The full search strategy can be found in S1 Table.

### Eligibility criteria and information sources

We included all studies (observational and experimental) reporting on successful policy initiatives aligned to UNAIDS targets, and socio-demographic variables. We classified eligible studies by type of intervention, study population, UNAIDS 90-90-90 targets [knowledge of HIV status from testing and diagnosis, initiation of antiretroviral therapy (ART), and viral suppression], country income level (low, middle, high), and reported outcomes. The country income strata were defined based on the 2016 World Bank Income Rank [10]. We excluded modeling papers, reviews, narratives, trial protocols and papers that were in a language other than English. As well, we explored bibliographies of articles for additional articles.

### Study selection

Three reviewers (NK, CF, and SD) independently screened, evaluated studies for eligibility and assessed the quality of included studies.

### Data collection process

Three reviewers (NK, CF, and SD) independently abstracted data. We used a predesigned abstraction form to collect information on the author, study type, study population, intervention, HIV outcome measure, and metrics used to measure reported outcomes regarding the 90-90-90 targets.

### Narrative review

Due to heterogeneity in reporting of study outcomes, we conducted a narrative review. If interventions were specific in reporting on UNAIDS targets, we classified them as successful. If interventions were unclear in reporting on UNAIDS targets and the metrics were complex, we refrained from such an analyses.

We stratified countries by income level to allow the comparison of countries and contexts to tailor HIV policy initiatives to their resource levels, improving the practicality of new policies [10].

### Risk of bias and quality assessment

The quality of studies was assessed using the Newcastle-Ottawa quality assessment scale for the observational studies.

## Results

**Fig 1:**
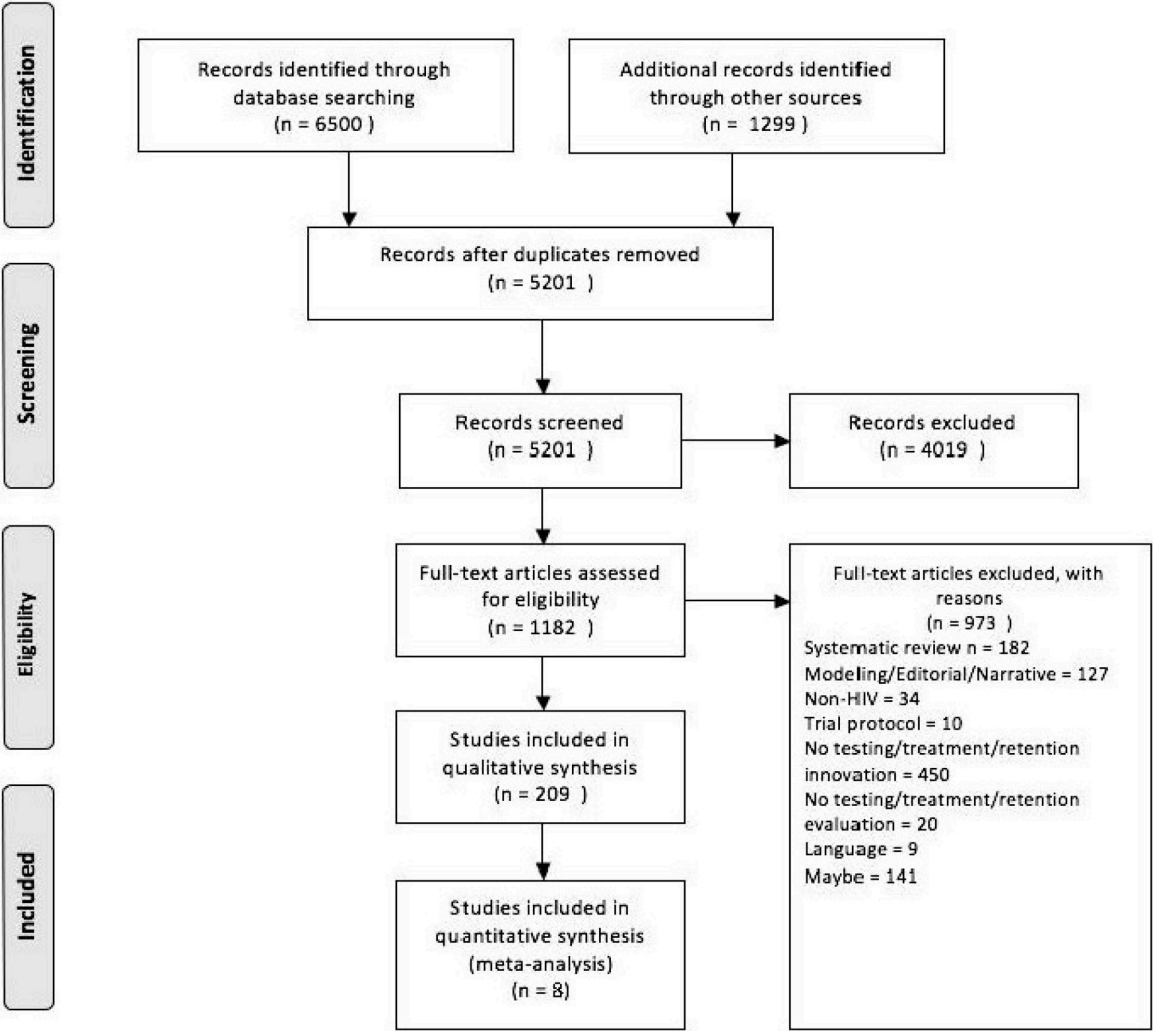
Preferred reporting items for systematic reviews and meta-analyses (PRISMA) flow chart.

We identified a total of 5201 citations. After applying our exclusion criteria, eight studies were included in the narrative synthesis. From these eight studies, eleven outcomes were identified (Fig 1).

### Study characteristics

Overall, 3/8 (37.5%) studies were from high-income countries (HICs), which included one from Canada and two from the United States. One (12.5%) study was from South Africa, a middle-income country (MIC). 4/8 (50%) studies were from low-income countries (LICs), specifically one from Malawi and three from Rwanda. All studies evaluated interventions but utilized different observational designs. These included five cross-sectional, two prospective, and one retrospective cohort study. 2/8 of the studies (25%) reported on more than one outcome, for a total of 11 reported outcomes (Table 1).

**Table 1:** Summary of study characteristics.

Country	Income Level	Measure/Metric	Intervention Type	Met target (✓) or Did not meet target (X)
USA^11^	HIC	HIV diagnosis	Expansive partner services policy	✓
USA^12^	HIC	Initiation into Care	Expanded HIV treatment policy	✓
Canada^13^	HIC	HIV diagnosis	Expanded partner notification	✓
South Africa^14^	MIC	Initiation to Care	Same-day diagnosis CD4 count guidelines	✓
Rwanda^15^	LIC	Initiation to Care	National HIV program	**X**
		Achieving Viral Suppression		✓
Rwanda^16^	LIC	Initiation to Care	ART initiation policy	✓
Malawi^17^	LIC	HIV diagnosis	*Médecins Sans Frontières* related program	✓
		Initiation to Care		✓
		Achieving Viral Suppression		✓
Rwanda^18^	LIC	HIV diagnosis	Community-based health insurance policy and performance based financing	✓

HIC, high income country; LIC, low income country; MIC, moderate income country; USA, United States.

Stratified by UNAIDS targets, 4/11 (36.4%) reported on the 1^st^ 90 (knowledge of HIV status, through testing and diagnosis), 5/11 (45.4%) on the 2^nd^ 90 (ART initiation) and 2/11 (18.2%) on the 3^rd^ 90 (viral suppression).

### Overview of studies

The interventions from each study are detailed in S2 Table. In order to incorporate income, a key driver of policies and programs, in the analysis, we further evaluated the studies by stratification into country income level.

This allowed for a more accurate and personalized assessment of factors supporting implementation, and allowed for a logical evaluation and comparison of all studies.

#### High-income countries

High-income countries targeted comprehensive partner services policies and expanded HIV treatment policy in the USA [11, 12], and expanded partner notification policies in Canada [13].

An example of success from the USA was the revised sexually transmitted disease (STD) partner services implemented by the Washington State’s Health Department that reported an increase in HIV testing from 63% to 91% (p<0.001) among partner services recipients [11]. Washington State’s revised STD partner service involved HIV testing for all men who have sex with men with early syphilis, gonorrhoea or Chlamydia [11]. The diagnosis of STDs, especially syphilis, is a risk factor for testing HIV+ as well; therefore the revised partner services encouraged HIV testing among those presenting with other STDs. Per the US Centers for Disease Control and Prevention, HIV testing is recommended to be carried out for all presenting with STD evaluation or treatment. However, only a small proportion of people screened for STDs are also tested for HIV [11].

Furthermore, the release of the US-based National HIV/AIDS Strategy and the expanded HIV treatment guidelines resulted in an increased prevalence of early linkage to care, from 79% in 2008 to 83% in 2011 (p = 0.44) [12].

Similarly, from Edmonton, Canada, the increased use of expanded partner notification guidelines was successful, with 346 HIV+ cases interviewed, 243 cases providing information on their partners, with 305/346 partners located (88.2%) and tested for HIV [13].

#### Middle-income countries

Policy guidelines for a same-day CD4 diagnosis were the target of a moderately successful intervention in South Africa [14]. Same-day CD4 count allowed for quicker ART initiation and linkage to care. This demonstrated that the implementation of the same-day diagnosis program led to the linkage of 75% of participants to care, whereas only 55% who did not receive same-day diagnosis blood draw were linked to care.

Their program illustrated that performing the blood draw immediately allowed for a significant increase in the number of patients (20%) linked to care. Although CD4 staging was replaced by viral load monitoring, in some cities, where CD4 testing is not phased out, this approach may be relevant.

#### Low-income countries

Low-income country interventions included three policies: a) National HIV program in Rwanda [15, 16], b) an ART program policy in Malawi [17], and c) community-based health insurance policies in Rwanda [18].

In a study by Ribakare et al., investigators aimed to quantify the HIV continuum of care in Rwanda by collecting data on individuals who participated in the national HIV care programme; 176,174/204,899 (86%) were in pre-ART or ART stages, while 129,405 individuals had initiated ART by 2013. Furthermore, 82.1% of HIV+ patients were virally suppressed [15]The Rwandan National Program employed a multi-sectoral response where they evaluated specific issues within their care cascade and created targets accordingly. The primary focus was to scale up HIV services by setting specific goals with measurable outcomes [15]. Other low-income countries can have similar successes so long as they focus resources to the appropriate targets and outcomes they see fit for their situation. This could mean first setting short-term goals to achieve eventual long-term goals. The Rwandan National Program also enabled an ART adherence rate of 82.1% [15].

As well, the Rwandan National HIV program demonstrated low attrition and higher linkage to care; 86.4% of people were linked to care in 2014 following the program’s implementation, as compared to 63% in 2013 [16].

A Malawian study evaluated the ART program supported by Médecins Sans Frontières, revealing that 7,270/8,277 (87.8%) participants were tested for HIV; of those that were HIV+ (1,233/7,270 participants), 71.2% were under care, 65.8% had initiated ART, and 61.8% were virally suppressed (viral load<1000 copies/mL). This study reported on all three UNAIDS targets, and reported a 72.8% ART adherence [17].

Another Rwandan study evaluated the impact community-based health insurance and performance-based financing programs had on health centers in Rwanda that provide key HIV services. Community-based health insurance increased community involvement, which assisted the centers’ ability to provide voluntary counselling and testing, as well as services for the prevention of mother-to-child transmission (PMTCT). Furthermore, centers that were incentivized by performance-based financing were also able to improve upon PMTCT [18]. Both Rwanda and Malawi are thus great models for other countries of similar economic status.

### Quality

Overall, since all above-described studies were observational in nature, biases exist namely confounding, selection and information. Two studies [13, 18] had a small sample size raising a potential for selection bias. Three studies [12, 14, 17] raised concern for outcome/exposure misclassification as their primary method of data collection was through self-reporting (Table 2).

**Table 2:** Quality assessment summary.

Author	Selection bias	Attrition bias	Confounding	Outcome/exposure misclassification	Comments
**Hoots, 2015**	Low	Low	Unclear	High	Outcome: Self-reporting
**Hoffman, 2016**	Low	Low	Unclear	High	Outcome: Self-reporting
**Katz, 2016**	Unclear	Low	High	Unclear	Confounding: testing occurred before PS interviews
**Ribakare, 2014**	Low	Low	Unclear	Unclear	N/A
**Maman, 2016**	Low	High	Unclear	High	Attrition: non-response to survey, biased estimates; Outcome: self-reported
**Zeng, 2014**	High	Low	Unclear	Unclear	Selection bias: small sample size
**Bergman, 2015**	High	Low	Unclear	Unclear	Selection bias: small sample size
**Nuwagaba-Biribonwoha, 2015**	Low	Low	Unclear	Unclear	N/A

## Discussion

### High-income countries (HIC)

In these settings, expanded partner notification programs/services and policies were shown to help increase the number of individuals being tested, thereby increasing the number of people living with HIV aware of their status and making progress towards the first target. These strategies suggest that more importance placed on the power of partner notification in similar settings can help to achieve 90% testing rates. However, partner notification programs that reported successes with ART initiation or linkages to care were not highlighted, suggesting that additional attention is required to showcase successful programs in these areas.

In regard to the second target in HIC, the success of the National HIV/AIDS Strategy and expanded HIV treatment guidelines in the USA highlighted the need to evaluate three parameters:

- Reduce the time between HIV diagnosis and linkage to care,
- Increase the proportion of virally suppressed at risk sub populations, and
- Significantly increase the number of individuals on ART [12].

Implementation of a policy also requires infrastructural changes. For instance, part of the success of the US national strategy is attributed to the Affordable Care Act. This Act requires that insurance coverage includes HIV testing and direct linkages to care. Furthermore, HIV positive men who have sex with men are eligible for further coverage to cover costs of treatment.

The studies in our review from high income countries did not capture policies or national programs targeting viral suppression or retention in care. However, evidence reported elsewhere has shown that HICs such as Sweden, Denmark, and the United Kingdom have exceeded the third target proportionally (reaching 73% viral suppression) with estimates that reported 78%, 80%, and 78% respectively. The Netherlands and Switzerland at 72%, are close to achieving 73% viral suppression target as well [19]. Three countries that have met these goals each have a publicly funded universal health care system, which potentially plays a major role in achieving the targets. Sweden, in particular, also implemented an education program to educate people who were diagnosed with HIV. Furthermore, HIV-positive individuals are required to disclose their HIV status to any sexual partners or they risk imprisonment. This is not to say that this method would be effective in all high-income countries seeing as cultural contexts are entirely different across countries, though, Sweden’s population is known to be egalitarian. In countries with a comparable cultural belief system, perhaps similar policies would be promising.

### Middle-income countries (MIC)

In MICs, strategies that improve HIV testing and linkage to care across the population spectrum have been prominent. A South African evaluation of the impact of same-day-diagnosis via the assessment of testers’ CD4 count revealed this policy’s ability to improve linkages to care [14]. This could as well be adapted for viral load based monitoring and adherence/retention measurements.

Of note, MICs such as Romania, Kazakhstan, and Thailand, have achieved successful testing rates of 98%, 92% and 89%, respectively [19]. In all these countries, advocacy has been a significant component of their policy implementations. Specifically, in Kazakhstan, policies that focus on collaborations with providers to minimize consequences associated with disclosure of sero-status are in place [20]. In Thailand, a program called “100% Condom Program” was introduced in 1991. It aims to conduct information campaigns and provide condoms to all illegal sex workers; rather than stigmatize them, the program sought to provide them with avenues to practice safe sex [21]. Furthermore, these three middle-income countries (Romania, Kazakhstan, Thailand) all have a universal healthcare system. It might be significant to consider the successes while keeping this context in mind. Effective MIC initiatives are similar to the efforts the U.S. President's Emergency Plan for AIDS Relief (PEPFAR) has encouraged in many African countries. PEPFAR aims to implement a Universal Test and Treat policy in order to rapidly expand ART to all people living with HIV. These efforts have shown great potential and findings from studies such as these further strengthen the positive direction we are heading towards achieving the targets by 2020.

### Low-income countries (LIC)

LICs require concerted efforts regarding all three aspects, namely, HIV testing, linkage and retention in care. Although testing rates have increased, successes in linkages to care remain a distant dream. Malawi and Rwanda have demonstrated successes in testing rates through implementation of a policy for decentralized testing [18]. This allows for small but densely populated countries to have better access to testing and care. This approach shows tremendous promise and should be implemented in neighbouring countries.

The Rwandan National Program employed a multi-sectoral response where they looked at specific issues within their care cascade and created targets accordingly. Their primary focus was to scale up HIV services by establishing specific goals with measurable outcomes [15].

Similarly, other LICs can experience successes comparable to Rwanda and Malawi, but they should be able to target their resources to achieve outcomes and targets as they see fit to their situation. This could mean setting short-term goals to achieve eventual long-term goals. The PEPFAR initiative has put in place an excellent policy plan for LICs, particularly with the Rwandan government where they are promoting their Test and Start ART policy.

### Caveats

Interestingly, the number of papers that reported country-level programs and policies were few. Although only eight interventions have come to light during our review of the literature, it is imperative to learn from these since they highlight innovative ways to engage people living with HIV into the cascade of care in its entirety.

The UNAIDS’ National Commitments and Policies Instrument (NCPI) is a questionnaire designed to keep track of HIV/AIDS policies and programs implemented or in development in 115 countries. While there are indeed many policies in place, there remains a lack in procuring data in published peer reviewed literature. Despite our exhaustive search, we could only find a few studies with good data on policies. Additionally, studies from many of the countries from the UNAIDS NCPI database were likely not captured by our search due to our eligibility criteria for the review. The UNAIDS database is not a traditional academic search database, hence it was excluded from our search.

This review offers a snapshot of successful programs and we plan to follow up on an extensive analyses of UNAIDS database alone, as a second step. We also propose to collect original data of programs/policies in future reviews.

There are a number of factors that determine the success of policy and the mere existence of policies does not guarantee success. The factors outlined by UNAIDS analyses that determine success in controlling the HIV epidemic hold true; yet the stage of epidemic in a country, the incidence/prevalence in affected populations, targeted programs that benefit these populations with engagement of populations in their own care is key to the success of these targets and eventually towards HIV control and elimination. Besides, the deployment of targets cannot be blinded; we need sensitization towards a standardized reporting of outcomes aligned to the targets, and clear explanations of which outcomes/metrics generate clear data for the targets, and that distinction is often not clear. Hence reporting is sometimes misleading and ineffectual.

The basic premise of this review is that in the identified countries under review, critical policies were created, implemented and enforced, and this lead to successful programs.

There are some caveats to consider while interpreting these findings. Across income levels, the literature has made it evident that a multipronged approach connecting programs and stakeholders via expanded testing, linkage, and retention in care services, is necessary. These programs have to be nested within the social or private health care systems in place in any country. Funded programs that are well supported by local governments have shown greater success and sustainability and have led to control of HIV. Although it seems clear that policies that enforce same-day diagnosis help improve linkage to care, it is essential to temper all these findings with caution. For linkage and retention metrics are two separate entities, and for control and elimination we need to have data on both to understand the reality of the HIV epidemic in the country.

A common theme of success across countries of all income levels was implementation of policies. In high-income countries, policies were enforced and followed, demonstrating in all the studies we evaluated, the UNAIDS 90-90-90 targets were met. In order to meet the targets, it was key for their policies to be well documented, have the required infrastructure, and be innovative at each step of the cascade of care. By extrapolation to low-income countries, our study would suggest that programmatic and policy implementations by organizations like Médecins Sans Frontières or by governments have the potential to be successful, provided certain conditions are in place to optimize their success.

In particular, the successful policy interventions of Rwanda and Malawi should be exemplified and could serve as a model for a successful implementation across similar low-income settings. Improved success rates would involve setting measurable shorter-term goals to eventually build to the achievement of long-term goals that have been set to a similar level to those of high-income countries.

An analysis of these studies also suggests that policies, combined with the scale-up incentives, are needed to change the status quo. Incentives to improve the targets must exist; performance incentives at the health care worker level, and country level incentives that could transform the nature of care.

### Conclusion

From the studies identified, we deduced a clear, positive correlation between implementation of policies and programs that resulted in an increase in patient awareness and an increase in partner notification with services that encouraged them, and together these resulted in increasing testing rates, and deployment of linkage/retention programs that improved retention in care.

Interestingly, we did not observe a discrepancy in the uptake of programs in countries with different incomes. Three studies, one from a HIC (USA) and two from LICs (Malawi and Rwanda), assessing linkages to treatment, demonstrated that regardless of income status, targeted programs, when applied or introduced, led to an increase in participants linked to care [12, 15, 17]. Perhaps, focused programs aimed at targets could work, because well-documented studies with innovative solutions for each target met the targets. Policies that enforced same-day diagnosis and facilitated linkage to care were also successful.

Overall, program reporting aligned to UNAIDS 90-90-90 targets were successful in demonstrating linkage of participants to care, thereby increasing the success rate of achieving the targets. Baggaley et al. have recently reported similar findings, stating that in order to conduct an appropriate global HIV response, prevention has to be re-focused [22].

Our review addresses policy and programmatic aspects, but also delves into the fact that the issue also perhaps lies in the reporting of outcomes. This does not imply that standardization of reporting is a concern but that there is an issue with the actual reporting. These facts are suggestive that there may in fact be successes in many countries that are not being reported. Sometimes outcomes and metrics that are not aligned to targets could lead to misclassification in reporting.

We conclude that there is an important need to identify key players, which include care givers, and provide them with support to capture the data, self-evaluate, and understand reporting of outcomes, metrics, and targets. A task force that customizes unique methods/policies, and capitalizes on the use of digital data and optimizes technologies through smartphones and web platforms could improve reporting on targets. This review makes us reflect that a “one size fits all” solution will not work and minor customizations will be important for success in each country. Implementation is essential, though monitoring is equally important to establish the country and state/province-specific interventions that are effective within each country/region. We propose that metrics such as those deployed by the President’s Emergency Plan for AIDS Relief Planning and Monitoring model be adapted and introduced to care givers in other countries [23]. This will result in an improved data collection and monitoring, while also help in expanding our understanding of successful programs. The successes previously observed with policy interventions provide an excellent framework and help us step in the right direction during the final push leading up to the UNAIDS 90-90-90 goal for 2020. Across all income levels, it is evident that a multipronged approach that integrates the cascade of care is urgently needed.

## Supporting information

S1 Checklist PRISMA checklist. (DOC)

S1 Table Search string for each database. (DOCX)

S2 Table Detailed characteristics of eligible studies. (DOCX)
