# Supplementary material for: Are policy initiatives aligned to meet UNAIDS 90-90-90 targets impacting HIV testing and linkages to care? Evidence from a systematic review
Source: PLoS One. 2019 Jun 5;14(6):e0216936. doi: 10.1371/journal.pone.0216936 (PMC6550376; doi:10.1371/journal.pone.0216936)
Supplement: S1 Table — (DOCX) [file pone.0216936.s002.docx]

**Search string for each database**

| **Database** | **Search String** |
| --- | --- |
| Biosis | ((("HIV" OR ("acquired immunodeficiency syndrome" OR "aids")) OR (“UNAIDS” OR “90-90-90”)) AND (“youth” OR “adults”) AND “viral load”) |
| CINAHL | (("HIV" OR "acquired immunodeficiency syndrome" OR "aids") OR (“UNAIDS” OR “90-90-90”)) AND (“youth” OR “adults” OR “viral load”) |
| Cochrane DARE | (HIV OR acquired immunodeficiency syndrome OR aids OR UNAIDS OR 90-90-90) AND (youth OR adults OR viral load) |
| EMBASE | (((HIV and acquired immunodeficiency syndrome and aids) or (UNAIDS and 90-90-90)) and (youth or adults or viral load)) |
| Global Index Medicus | (hiv OR UNAIDS) AND (infants OR adults OR viral load) |
| LILACS | (HIV OR acquired immunodeficiency syndrome OR aids OR UNAIDS OR 90-90-90) AND (youth OR adults OR viral load) |
| OVID | ((HIV and acquired immunodeficiency syndrome and aids) or (UNAIDS and 90-90-90)) and ((youth or adults) or (viral load)) |
| PubMed | (("HIV"[MeSH Terms] OR "HIV"[All Fields]) OR ("acquired immunodeficiency syndrome"[MeSH Terms] OR ("acquired"[All Fields] AND "immunodeficiency"[All Fields] AND "syndrome"[All Fields]) OR "acquired immunodeficiency syndrome"[All Fields] OR "aids"[All Fields])) AND (“UNAIDS”[All Fields] AND/OR “90-90-90”[All Fields] AND/OR (“youth” OR “adults”) AND “viral load”) |
