# Supplementary material for: Are policy initiatives aligned to meet UNAIDS 90-90-90 targets impacting HIV testing and linkages to care? Evidence from a systematic review
Source: PLoS One. 2019 Jun 5;14(6):e0216936. doi: 10.1371/journal.pone.0216936 (PMC6550376; doi:10.1371/journal.pone.0216936)
Supplement: S2 Table — (DOCX) [file pone.0216936.s003.docx]

**Detailed Characteristics of Eligible Studies** (MSM = men who have sex with men; ARV = antiretroviral; ART = antiretroviral therapy; 95% CI when presented; ATT = attendance)

| **Author** | **Study Design** | **Participants/ Country** | **Intervention** | **Measure/Metric** | **Results** | **90-90-90 Target** |
| --- | --- | --- | --- | --- | --- | --- |
| **Hoots, 2015** | Cross-sectional | ≥18 yrs, MSM  USA  n = 1144 (2008)  n =1338 (2011) | Assessing the impact of National HIV/AIDS Strategy and expanded HIV treatment guidelines via participant interviews on ARV treatment and linkage to care among MSM | ATT treatment: linked to care | Prevalence of early linkage to care was 79% (187/236) in 2008 and 83% (241/291) in 2011. In multivariable analysis, prevalence of early linkage did not differ significantly between years overall (p=0.44). | 2^nd^ |
|  |  |  |  | ATT treatment: self-reported | Prevalence of ARV treatment was 69% (790/1,142) in 2008 and 79% (1,049/1,336) in 2001. In multivariable analysis, ARV treatment increased overall (p=0.0003) and among most sub-groups. Black MSM were less likely than white MSM to report ARV treatment (p= 0.01). / Effective. | 2^nd^ |
| **Hoffman, 2016** | Prospective cohort study | ≥18 yrs, HIV+  South Africa  n=459 | Evaluated the impact of not having a day-of-diagnosis CD4+ count blood draw, as recommended by South African guidelines, on time to linkage. Newly-diagnosed participants were followed for 8M with three structured assessments. Linkage to care, defined as returning to clinic for CD4+ count results, and day-of-diagnosis blood draw were self-reported | ATT treatment: linked to care | Newly-diagnosed HIV+ individuals who did not undergo CD4+ count blood draw on the day they were diagnosed had delayed linkage to care relative to those with same-day blood draw. 72.5% did not have a day-of-diagnosis CD4+ count blood draw, and 19.2% of these never returned. Compared with a day-of-diagnosis blood draw, the adjusted hazard ratio of linkage (AHR linkage) associated with not having day-of-diagnosis blood draw was 0.66 (95%CI: 0.51, 0.85). By 4 months, 54.8% of those without day-of-diagnosis blood draw vs. 75.2% with one were linked to care (chi-squared p = 0.004). / Effective. | 2^nd^ |
| **Katz, 2016** | Prospective cohort study | MSM with early syphilis, gonorrhea, or Chlamydia  USA  n = 8133 | Health departments in Washington State revised STD partner services programs to provide partner services to all MSM with early syphilis (primary, secondary, or early latent), gonorrhea, or chlamydial infection to ensure that all MSM and sex partners without a prior HIV diagnosis were tested for HIV infection before closure of the index case. | ATT testing: Attendance rate | Implementation of the intervention was associated with an increase in HIV testing among MSM without a prior HIV diagnosis who received PS (63 to 91%, p < 0.001). Providers were more likely to test MSM for HIV infection at the time of STD diagnosis or treatment before and during the intervention periods (p < 0.001). | 1^st^ |
| **Nuwagaba-Biribonwoha, 2014** | Retrospective cohort study | ≥18 yrs, HIV+, CD4<200 cells/uL  Rwanda  n = 1678 | Determination of attrition in Rwandan National Program for those initiating ART 6, 12 and 18 months prior to study | ATT treatment: LTFU or death | High levels of retention on ART were reported for the first few years of the national program. The total amount of follow-up time was 1508 person years, while the average follow-up time was 11.3 months. The weighted population was 8,373, 3123 [37.3%], 2762 [33.0%], and 2488 [29.7%] patients who had initiated ART 6, 12 and 18 months prior to data collection respectively, and represented 86.4% of the sampling frame population of 9,693. | 2^nd^ |
| **Maman, 2016** | Cross-sectional | 15-59 yrs  Malawi  n = 7269 | The antiretroviral therapy programme supported by *Medecins Sans Frontieres* in the rural Malawian district of Chiradzulu. Participants completed an individual questionnaire and provided blood for the HIV test | ATT testing: Attendance rate | 7269 (87.9%) of the eligible 8271 participants tested for HIV, 1233 were found to be HIV positive. The overall weighted prevalence was 17.0% (95% CI 16.1 to 17.9). Prevalence was higher for women than men (19.7 vs. 13.0, p B0.01). | 1^st^ |
|  |  |  |  | ATT treatment: linked to care (self-reported) | The proportion of the HIV-positive individuals ever linked to care was 74.2% (95% CI 72.5 to 76.7). Retention in care was also high at 72.8% (95% CI 70.1 to 75.3) | 2^nd^, 3^rd^ |
|  |  |  |  | Feasibility: Completion rate | Of the 1233 HIV+ participants, a total of 1174 (95.1%) had completed the individual questionnaire and had their CD4 cell count and HIV load ascertained. Among them, 77.0% (95% CI 74.4 to 79.3) reported being already diagnosed for HIV | Other |
| **Zeng, 2014** | Descriptive | Rural health centers providing ART services in Rwanda  Health centers  n=26 (6 that started ART 2003-2005, 20 that started ART in 2006) | Effect of Performance based financing (PBF) and community-based health insurance (CBHI) policies implemented by NGOs among health centers in Rwanda over 2002-2006 paid organizations based on volume and quality of services. | Testing services offered | CBHI use rate improved prevention of mother-to-child transmission (PMTCT) (coefficient 0.037, p value=0.05) and voluntary counseling and testing (coefficient 0.025, p value=0.10), PBF increased PMTCT services by 87% (p value=0.10) | 1^st^ |
| **Bergman, 2015** | Descriptive | HIV+  Edmonton, Alberta, Canada  n=346 | HIV positive results were followed up by a  partner notification (PN) nurse to conduct PN for all sexual, injection drug user (IDU), and peri-natal contacts within the previous 6 months prior to the last negative HIV test or the estimated date of sero-conversion | Partner notification: proportion of partners notified | Seventy percent (n = 243) of the index cases provided partner information. cases that did not provide partners  were older (41 years, IQR 33–51 vs. 35 years, IQR 28–44;  p < 0.001) and were more likely to have been exposed through nonsexual and non-IDU routes (88.9% (n = 8) vs. 28.2% (n = 95); p < 0.001). 305 new partners (88.2%) were tested, resulting in 20 new diagnoses of HIV | 1^st^ |
| **Ribakare, 2015** | Cross-sectional | HIV+  Rwanda  n=204,899 (estimate) | Rwanda national HIV programme | ATT treatment: follow-up appointment | 129 405 (63%) of HIV-positive Rwandans had initiated ART by the end of 2013. | 2^nd^ |
|  |  |  |  | ART adherence: CD4 | 82·1% (95% CI 80·7–83·4) of patients with viral load measurements (n=3066) were virally suppressed. | 3^rd^ |
